# Supplementary material for: Influence of Electronic Cigarette Characteristics on Susceptibility, Perceptions, and Abuse Liability Indices among Combustible Tobacco Cigarette Smokers and Non-Smokers
Source: Int J Environ Res Public Health. 2019 May 23;16(10):1825. doi: 10.3390/ijerph16101825 (PMC6572235; doi:10.3390/ijerph16101825)
Supplement: Supplementary file 1 [file ijerph-16-01825-s001.pdf]

Supplemental Figure S1: Example ECIG Regulatory Condition (No Nicotine) Outcome Measures

Susceptibility to ECIG Use (4 items)

Do you think that you will use an e-cigarette with no nicotine soon?

- Definitely yes
- Probably yes
- Probably not
- Definitely not

Do you think that in the future you might experiment with e-cigarettes with no nicotine? Would you say . . .

- Definitely yes
- Probably yes
- Probably not
- Definitely not

Do you think you will use an e-cigarette with no nicotine in the next year? Would you say. . .

- Definitely yes
- Probably yes
- Probably not
- Definitely not

If one of your best friends were to offer you an e-cigarette with no nicotine, would you use it? Would you say . . .

- Definitely yes
- Probably yes
- Probably not
- Definitely not

Perceived ECIG relative harm and addiction (2 items)

Compared to regular-strength non-menthol cigarettes, how harmful do you think that the following product is to your health?

E-cigarette with no nicotine

A lot less harmful	A little less harmful	About the same	A little more harmful	A lot more harmful	Don't know

What do you think the likelihood of addiction is when using each of these products?

E-cigarette with no nicotine

Not at all	Slightly	Moderately	Very Much	Extremely	Don't know

ECIG Purchase Task (16 prices)

E-cigarette No Nicotine Purchasing Task

Imagine a TYPICAL DAY. The following questions ask how many times you would take 10 puffs of an **e-cigarette with no nicotine** if every 10 puffs cost various amounts of money. **The only available e-cigarette is the one with no nicotine**. Assume that you have the same income/savings that you have now and NO ACCESS to any other tobacco or nicotine products other than the e-cigarette offered at these prices. In addition, assume that you would consume the e-cigarette puffs that you request on that day; that is, you cannot save or stockpile e-cigarette puffs for a later date. Please respond to these questions honestly.

How many times would you take 10 puffs of an **e-cigarette with no nicotine** if they were ____each at the following prices?

	Number of times you would take 10 puffs of an e-cigarette with no nicotine
Price per 10 puffs of an e-cigarette with no nicotine: $0 (free)	
Price per 10 puffs of an e-cigarette with no nicotine: $0.01	
Price per 10 puffs of an e-cigarette with no nicotine: $0.02	

Price per 10 puffs of an e-cigarette with no nicotine: $0.04	
Price per 10 puffs of an e-cigarette with no nicotine: $0.08	
Price per 10 puffs of an e-cigarette with no nicotine: $0.16	
Price per 10 puffs of an e-cigarette with no nicotine: $0.32	
Price per 10 puffs of an e-cigarette with no nicotine: $0.64	
Price per 10 puffs of an e-cigarette with no nicotine: $1.28	
Price per 10 puffs of an e-cigarette with no nicotine: $2.56	
Price per 10 puffs of an e-cigarette with no nicotine: $3.84	

Price per 10 puffs of an e-cigarette with no nicotine: $5.12	
Price per 10 puffs of an e-cigarette with no nicotine: $6.40	
Price per 10 puffs of an e-cigarette with no nicotine: $7.68	
Price per 10 puffs of an e-cigarette with no nicotine: $8.96	
Price per 10 puffs of an e-cigarette with no nicotine: $10.24	

Supplemental Table S1. Adjusted Associations for All Covariates among the Nicotine Domain and Susceptibility, Perceptions, and Abuse Liability Indices.

	Susceptibility to ECIG use (n=186)		Perceived ECIG Relative Harm (n=245)		Perceived ECIG Addiction (n=245)		Log-Breakpoint (n=254)		Log-Intensity (n=254)	
Variable	AOR	*p*	β	*p*	β	*p*	β	*p*	β	*p*
ECIG Regulatory Condition										
No nicotine ECIG	Ref		Ref		Ref		Ref		Ref	
Low nicotine content ECIG	2.22	0.098	**0.42**	**0.006**	**1.14**	**<0.001**	**0.69**	**0.034**	0.72	0.107
High nicotine content ECIG	1.60	0.341	**1.19**	**<0.001**	**1.96**	**<0.001**	0.50	0.143	0.33	0.479
Gender										
Male	Ref		Ref		Ref		Ref		Ref	
Female	0.66	0.302	-0.09	0.468	-0.06	0.603	-0.11	0.703	-0.15	0.696
Age										
18-25	Ref		Ref		Ref		Ref		Ref	
26-29	1.34	0.654	-0.39	0.057	-0.18	0.352	-0.25	0.571	-0.54	0.378
30-36	0.74	0.638	-0.15	0.454	0.16	0.370	-0.10	0.810	-0.65	0.271
37+	0.74	0.613	-0.11	0.575	**0.44**	**0.013**	0.03	0.948	-0.07	0.894
Race/ethnicity										
White/Caucasian	Ref		Ref		Ref		Ref		Ref	
Asian	0.65	0.576	0.45	0.070	-0.07	0.770	0.80	0.143	0.05	0.943
Other	0.45	0.170	**0.50**	**0.007**	0.18	0.281	0.19	0.621	-0.38	0.469
Education										
High School/GED or below	Ref		Ref		Ref		Ref		Ref	
Some College	1.39	0.581	-0.02	0.914	-0.13	0.429	0.29	0.449	-0.09	0.862
College Graduate	1.60	0.447	0.04	0.839	-0.05	0.783	0.40	0.306	-0.09	0.867
Post-college Education	3.45	0.120	0.10	0.678	-0.11	0.649	0.86	0.114	0.05	0.943
CTC/ECIG Status										
Non-CTC/ECIG user	Ref		Ref		Ref		Ref		Ref	
CTC-only smoker	**29.75**	**<0.001**	-0.26	0.097	-0.06	0.674	2.94	**<0.001**	4.14	**<0.001**
Dual CTC/ECIG user	-[a]	-[a]	**-0.47**	**0.005**	-0.26	0.087	3.62	**<0.001**	5.59	**<0.001**

Note: CTC= Combustible Tobacco Cigarette; ECIG= Electronic Cigarette; GED=General Education Diploma; AOR= Adjusted Odds Ratio; Ref = reference category. **Bold** values indicate statistical significance ($p<0.05$). [a] Due to cell size frequency, this response category was not included in analyses for the susceptibility to ECIG use outcome.

Supplemental Table S2. Adjusted Associations for All Covariates among the ECIG Flavor Domain and Susceptibility, Perceptions, and Abuse Liability Indices

	Susceptibility for ECIG use (n=184)		Perceived ECIG Relative Harm (n=247)		Perceived ECIG Addiction (n=247)		Log-Breakpoint (n=261)		Log-Intensity (n=261)	
	AOR	*p*	β	*p*	β	*p*	β	*p*	β	*p*
ECIG Regulatory Condition										
Tobacco-flavored ECIG	Ref		Ref		Ref		Ref		Ref	
Menthol-flavored ECIG	**0.33**	**0.020**	**0.52**	**0.001**	0.25	0.136	**-0.93**	**0.008**	**-1.33**	**0.002**
Fruit-flavored ECIG	0.77	0.548	0.08	0.603	-0.09	0.597	-0.15	0.663	0.21	0.612
Gender										
Male	Ref		Ref		Ref		Ref		Ref	
Female	0.66	0.264	-0.13	0.265	0.00	0.983	0.39	0.168	0.31	0.384
Age										
18-25	Ref		Ref		Ref		Ref		Ref	
26-29	1.14	0.808	-0.12	0.490	-0.01	0.979	-0.06	0.891	0.47	0.371
30-36	0.49	0.215	0.01	0.967	0.23	0.280	-0.40	0.351	-0.36	0.503
37+	0.51	0.226	-0.08	0.671	0.07	0.735	0.11	0.803	-0.44	0.406
Race/ethnicity										
White/Caucasian	Ref		Ref		Ref		Ref		Ref	
Asian	0.53	0.443	**0.60**	**0.016**	-0.02	0.937	-0.71	0.229	-0.46	0.529
Other	1.00	0.995	0.09	0.580	-0.35	0.068	0.45	0.254	0.48	0.321
Education										
High School/GED or below	Ref		Ref		Ref		Ref		Ref	
Some College	1.38	0.584	-0.05	0.799	0.26	0.260	0.28	0.547	0.40	0.487
College Graduate	1.34	0.592	0.07	0.727	0.05	0.817	0.14	0.748	-0.16	0.776
Post-college Education	0.49	0.379	0.21	0.384	0.14	0.617	-0.78	0.152	-0.93	0.171
CTC/ECIG Status										
Non-CTC/ECIG user	Ref		Ref		Ref		Ref		Ref	
CTC-only smoker	9.55	**<0.001**	**0.41**	**0.004**	0.10	0.546	2.31	**<0.001**	3.76	**<0.001**
Dual CTC/ECIG user	-[a]	-[a]	-0.16	0.259	0.02	0.925	3.41	**<0.001**	5.47	**<0.001**

Note: CTC= Combustible Tobacco Cigarette; ECIG=e-cigarette; GED=general education diploma; AOR= Adjusted Odds Ratio**; Bold** values indicate statistical significance (p<0.05). [a] Due to cell size frequency, this response category was not included in analyses for the susceptibility to ECIG use outcome

Supplemental Table S3. Adjusted Associations for All Covariates among the Modified Risk Message Domain and Susceptibility, Perceptions, and Abuse Liability Indices

	Susceptibility to ECIG use (n=138)		Perceived ECIG Relative Harm (n=177)		Perceived ECIG Addiction (n=174)		Log-Breakpoint (n=184)		Log-Intensity (n=184)	
	AOR	*p*	β	*p*	β	*p*	β	*p*	β	*p*
ECIG Regulatory Condition										
Reduced harm ECIG	Ref		Ref		Ref		Ref		Ref	
Reduced CE ECIG	0.40	0.075	0.16	0.173	0.17	0.232	0.19	0.542	-0.41	0.255
Gender										
Male	Ref		Ref		Ref		Ref		Ref	
Female	**0.27**	**0.014**	**0.28**	**0.021**	0.16	0.258	-0.32	0.311	-0.44	0.230
Age										
18-25	Ref		Ref		Ref		Ref		Ref	
26-29	0.83	0.798	-0.13	0.477	0.22	0.295	0.12	0.789	-0.06	0.918
30-36	0.49	0.360	0.06	0.712	0.30	0.141	**-1.08**	**0.019**	-0.60	0.258
37+	0.48	0.331	-0.16	0.348	0.28	0.176	-0.30	0.512	-0.26	0.626
Race/ethnicity										
White/Caucasian	Ref		Ref		Ref		Ref		Ref	
Asian	3.82	0.085	-0.20	0.291	**-0.47**	**0.039**	-0.59	0.236	0.01	0.991
Other	0.96	0.960	0.00	0.983	**-0.73**	**<0.001**	-0.72	0.113	**-1.33**	**0.012**
Education										
High School/GED or below	Ref		Ref		Ref		Ref		Ref	
Some College	0.52	0.506	0.02	0.916	-0.04	0.878	0.33	0.525	-0.08	0.895
College Graduate	0.97	0.976	-0.04	0.824	-0.18	0.454	0.51	0.333	-0.13	0.837
Post-college Education	1.35	0.808	-0.11	0.663	-0.02	0.952	1.07	0.102	-0.20	0.796
CTC/ECIG Status										
Non-CTC/ECIG user	Ref		Ref		Ref		Ref		Ref	
CTC-only smoker	**43.88**	**<0.001**	0.16	0.253	-0.23	0.170	3.71	**<0.001**	4.98	**<0.001**
Dual CTC/ECIG user	-[a]	-[a]	**-0.61**	**<0.001**	-0.19	0.284	4.19	**<0.001**	6.94	**<0.001**

Note: CE=carcinogen exposure; CTC=Combustible Tobacco Cigarette; ECIG=e-cigarette; GED=general education diploma; AOR= Adjusted Odds Ratio; **Bold** values indicate statistical significance (p<0.05).[a] Due to cell size frequency, this response category was not included in analyses for the susceptibility to ECIG use outcome.
